# Efficacy of bone marrow stimulation in arthroscopic repair of full thickness rotator cuff tears: a meta-analysis

**DOI:** 10.1186/s13018-019-1072-6

**Published:** 2019-01-29

**Authors:** Zhuoyang Li, Yijun Zhang

**Affiliations:** Department of Orthopedics, The First Affiliated Hospital, College of Medicine, Zhejiang University, Hangzhou, China

**Keywords:** Bone marrow stimulation, Rotator cuff repair, Tendon-to-bone healing, Meta-analysis, Biological therapy

## Abstract

**Background:**

To conduct a meta-analysis to compare the curative effect of treating the full thickness tear of the rotator cuff using the arthroscopic bone marrow stimulation (BMS) technology and provide the evidence for its extensive clinical application.

**Methods:**

A systematic literature search was conducted to evaluate the studies on comparison of the curative effect of routine surgery with or without BMS on rotator cuff tear in the major medical databases. The literature was screened according to the inclusion and exclusion standards, and the quality assessment was conducted, then Review Manager 5.3 software was used for meta-analysis.

**Results:**

Eight articles were eligible for inclusion. There were no statistically significant differences between BMS and control groups for overall outcome scores (*P* > 0.05). Except the Constant score of BMS group was significantly higher than that of the control group at the third follow-up month (*P* = 0.007). However, the postoperative re-tear rate of the BMS group was significantly lower than that of the control group (*P* < 0.001). Furthermore, we made a subgroup analysis and found that the postoperative Constant and UCLA score had no significant differences among all groups (*P* > 0.05), and the re-tear rates of the BMS groups were lower than those of the control groups (*P* = 0.001, *P* = 0.0002).

**Conclusions:**

BMS technology has no significant influence on the postoperative clinical result of patients. However, it can obviously promote the tendon-to-bone healing of the rotator cuff and decrease the re-tear rate, which provides evidence for the clinical treatment.

## Background

The rotator cuff tear is one of the common shoulder diseases. The incidence is rising with the increase of age, which could be approximately 50% of the over-60s and up to 80% of the over-80s [1]. Currently, arthroscopic surgery is the primary surgical treatment, with single-row or double-row anchor fixation suturing. The rotator cuff is composed of longitudinal collagen fiber and few blood vessels, leading to its poor self-repairing capability. While existing fixation methods are mostly making the tendon direct contact with bone, which results in the difficulty of healing, as lacking normal fibrocartilage structure, especially for the large-massive rotator cuff tear [2], literature reports that the re-tear rate of repaired large-massive rotator cuff tear is as high as 30–94% [3]. Thus, more and more scholars have paid great attention to the biological repair of tendon-to-bone interface instead of only fixing with anchor so as to increase the fixation intensity.

There are lots of reports on the biological repair technology promoting tendon-to-bone healing, such as the platelet-rich plasma (PRP), mesenchymal stem cells (MSCs), and autologous tendon cell transplantation [4, 5]. However, the efficacy of these methods is unsatisfactory, especially for the large-massive rotator cuff tear. Bryan et al. made a systematic evaluation on the PRP-related META analysis of recent years and found that the highest evidence showed applying the PRP during the surgery generally cannot improve the re-tear rate and clinical function compared with the control [6]. And some scholars considered that the adipose-derived MSCs or autologous tendon cells applied only to the small rotator cuff tear because of the too complicated operation [7, 8].

Bone marrow stimulation (BMS) technology of subchondral bone has been confirmed by many scholars and is supposed to promote the biological repair of cartilage defect of the knee or ankle joint [9, 10]. The “crimson duvet” was proposed to be applied to the shoulder rotator cuff repair by Snyder in 2009 for the first time, which could provide MSCs with the micro-fracture treatment on the footprint [11]. As a simple, secure, and effective biological repair technology, it has received increasing attention from scholars in recent years. More and more evidence in animal experiments showed that the overflowing MSCs to the tendon-to-bone interface could rebuild the fibrocartilage structure so as to increase the ultimate force to failure [12, 13]. However, recent researches had the inconsistent result: applying the BMS did not show any clinical advantage over the control group [14–16]. Thus, this study plans to make a meta-analysis on the related literature to evaluate whether the application of BMS in the rotator cuff neoplasty will impact the re-tear rate and the clinical consequence, which hopes to provide an evidence for the clinical treatment.

Our hypothesis was that BMS would significantly improve outcomes or re-tear rates of patients with arthroscopic rotator cuff repair when compared with controls.

## Methods

### Inclusion and exclusion criteria

#### Inclusion criteria

The inclusion criteria were as follows: (1) patients diagnosed with full thickness rotator cuff tears underwent the arthroscopic rotator cuff neoplasty and followed up for more than 12 months; (2) randomized controlled trial (RCT) and retrospective cohort study; (3) interventions: the control group was treated with the conventional surgery, such as single-row or double-row fixation, and the experimental group was treated with a combination of the BMS technique; (4) the study included clinical and imaging results of the treatment with or without BMS; and (5) observation indicators: clinical function scores, including the Constant score, the University of California at Los Angeles (UCLA) score, the American Shoulder and Elbow Surgeons (ASES) score, the Simple Shoulder Test (SST) score, visual analogue scale (VAS), the postoperative range of motion (ROM), the rate of re-tear, and other complications in both groups.

#### Exclusion criteria

The exclusion criteria were as followed: (1) interventions were not included in the above types, (2) follow-up time was less than 12 months, and (3) studies of repeated publications were excluded. Summaries, lectures, reviews, and case reports were also excluded.

### Retrieval strategies

This study searched PubMed, EMBASE, Springer, Ovid, Cochrane Library, China National Knowledge Infrastructure (CNKI), China Biology Medicine Disc (CBMdisc), and other medical literature databases for all the related articles published from January 1980 to April 2018 according to the Prima Guidelines recommended by the Cochrane Collaboration. The keywords were “rotator cuff,” “Bone marrow stimulation,”, “microfracture,” “BMSCs,” and so on.

### Data extraction and quality assessment

We extracted data by retrieving the following information: publication time, first author, study design, patient information, surgical plan, clinical function score, ROM, the re-tear rate, and so on.

RevMan software was used to evaluate the quality of the included studies. The parameters included sequence generation (selection bias), allocation hiding (selection bias), blindness (performance bias), incomplete result data (detection bias), selective result reporting (reporting bias), and “other issues.” Each item could be classified as “low risk,” “high risk,” or “unclear.” The quality of retrospective cohort studies was assessed by the Newcastle-Ottawa Scale (NOS). The two evaluators independently rated the quality of these studies. The differences were resolved by the third reviewer.

### Statistical analysis

The statistical analysis was performed using Review Manager 5.3 software (Cochrane Collaboration, Nordic Cochrane Centre, Copenhagen, Denmark). Continuous variables were analyzed using the weighted mean difference, and categorical variables were assessed using relative risks. *P* < 0.05 was considered to be statistically significant, and 95% CIs were reported. Homogeneity was tested by the *Q* statistical (significance level at *P* < 0.1) and the *I*^2^ statistical (significance level at *I*^2^ > 50%). When there was no significant statistical heterogeneity, the fixed effects model was used. Otherwise, the random effects model was used. Furthermore, we made the subgroup analysis of the Constant score, the UCLA score, and the re-tear rate.

## Results

### Literature retrieval results

**Fig. 1:**
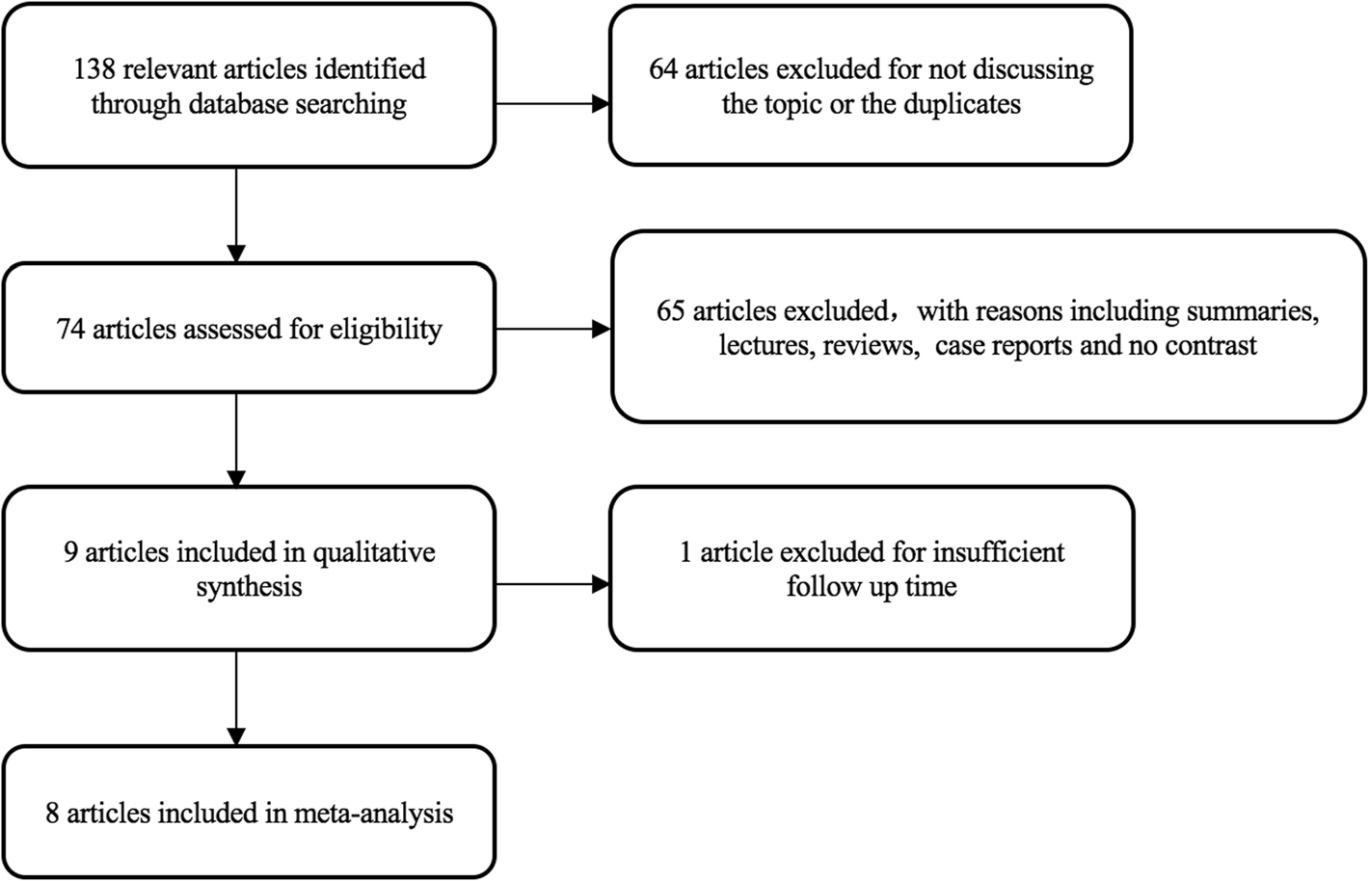
Search strategy flow diagram

One hundred thirty-eight articles consistent with the study purpose were identified, and after reading the full text, we chose 8 studies [14–21] including 4 RCTs and 4 retrospective cohort studies, with a total of 633 patients. The literature retrieval process is illustrated in Fig. 1. The basic characteristics of these studies are shown in Table 1. BMS preparation protocols of the included studies are shown in Table 2 [22].

**Table 1:** Demographic characteristics of the included studies

First author, year	Study design	Number		Age		Male/female		Operation methods	Outcome	Follow-up	NOS (4)
		BMS	Control	BMS	Control	BMS	Control				
Jo 2011	Retrospective cohort	25	31	58.3	56.5	11/14	15/16	Double-row repair with/without BMS	The incidence of re-tear, structural integrity	12 to 17 m	5
Jo 2013	Retrospective cohort	57	67	58.9	60.1	25/32	33/34	Double-row repair with/without BMS	The incidence of re-tear, Constant, UCLA, ROM, VAS, DASH, SST, SPADI, ASES, structural integrity	23 to 57 m	7
Milano 2013	RCT	35	38	60.6	63.1	22/13	19/19	Single-row repair with/without BMS	The incidence of re-tear, Constant, DASH, structural integrity	25 to 31 m	NA
Osti 2013	RCT	28	29	61.2	59.8	16/12	13/16	Single-row repair with/without BMS	The incidence of re-tear, Constant, UCLA, ROM	24 to 53 m	NA
Cai 2016	RCT	51	53	62.9	61.3	24/27	32/21	Double-row repair with/without BMS	The incidence of re-tear, Constant, UCLA	24 to 36 m	NA
Zhang 2016	RCT	20	20	58.6	59.5	9/11	14/6	Double-row repair with/without BMS	The incidence of re-tear, Constant, UCLA	12 m	NA
Taniguchi 2015	Retrospective cohort	44	67	64.7	64.3	22/15	42/25	Surface-holding with/without BMS	The incidence of re-tear, Structural integrity, complication	12 to 24 m	6
Yoon 2016	Retrospective cohort	21	54	64.9	62.8	9/12	26/28	Double-row repair with/without BMS	The incidence of re-tear, Constant, UCLA, VAS, SST, ASES, ROM	14 to 43 m	7

*RCT* randomized control study, *BMS* bone marrow stimulation, *UCLA* University of California at Los Angeles, *ROM* region of motion, *VAS* visual analogy score, *DASH* Disabilities of the Arm, Shoulder and Hand, *SST* Simple Shoulder Test, *SPADI* Shoulder Pain and Disability Index, *ASES* American Shoulder and Elbow Surgeons, Follow-up (months)

**Table 2:** Preparation protocols of bone marrow stimulation

First author, year	Instrument	Diameter (mm)	Interval (mm)	Depth (mm)	Location	Postoperative rehabilitation protocol				
						Immobilized	Passive ROM exercise	Active-assisted ROM exercise	Strengthening exercise	Full return to Sports
Jo 2011	Bone punch	2.1	4–5	10	From the articular cartilage margin to the lateral ridge of the greater tuberosity.	For 4–6 weeks using an abduction brace	The day after surgery for small to large size tear; from 6 weeks after surgery for massive tear	From 4 to 6 weeks after surgery	From 12 weeks after surgery	From 6 months after surgery
Jo 2013	Bone punch	2.1	4–5	10	From the articular cartilage margin to the lateral ridge of the greater tuberosity.	For 4–6 weeks using an abduction brace	The day after surgery for small to large size tear; from 6 weeks after surgery for massive tear	From 4 to 6 weeks after surgery	From 12 weeks after surgery	From 6 months after surgery
Milano 2013	arthroscopic awl	1.5	4	5	The attachment area of the tendons, along the articular margin	For 4 weeks using a sling	From 4 weeks after surgery according to the principles of shoulder rehabilitation program by Kibler [22]			
Osti 2013	Arthroscopic awl	Unknown	3–4	2–4	From the juxta-articular into the subacromial space	For 4 weeks using a sling	From 2 to 4 weeks after surgery	From 6 weeks after surgery	From 12 weeks after surgery	NA
Cai 2016	Lumbar puncture needle	0.5	2	3	Footprint region	For 6 weeks using an abduction brace	From 0 to 6 weeks after surgery	From 6 to 8 weeks after surgery	From 6 to 8 weeks after surgery	From 6 months after surgery
Zhang 2016	Lumbar puncture needle	0.5	2	3	Footprint region	For 4–6 weeks using an abduction brace	From 1 to 6 weeks after surgery	From 6 to 8 weeks after surgery	From 6 to 8 weeks after surgery	From 6 months after surgery
Taniguchi 2015	Metal bar	3.0	3–5	Unknown	Along the medially advanced footprint	For 6–8 weeks using an abduction pillow	From 2 weeks after surgery	From 8 to 10 weeks after surgery	From 10 to 12 weeks after surgery	From 6 months after surgery
Yoon 2016	Bone punch	2.1	4–5	10	From the articular cartilage margin to the lateral ridge of the greater tuberosity.	For 8 weeks using an abduction brace	From 8 weeks after surgery	From 8 weeks after surgery	From 12 weeks after surgery	From 6 months after surgery

### Literature quality assessments

**Fig. 2:**
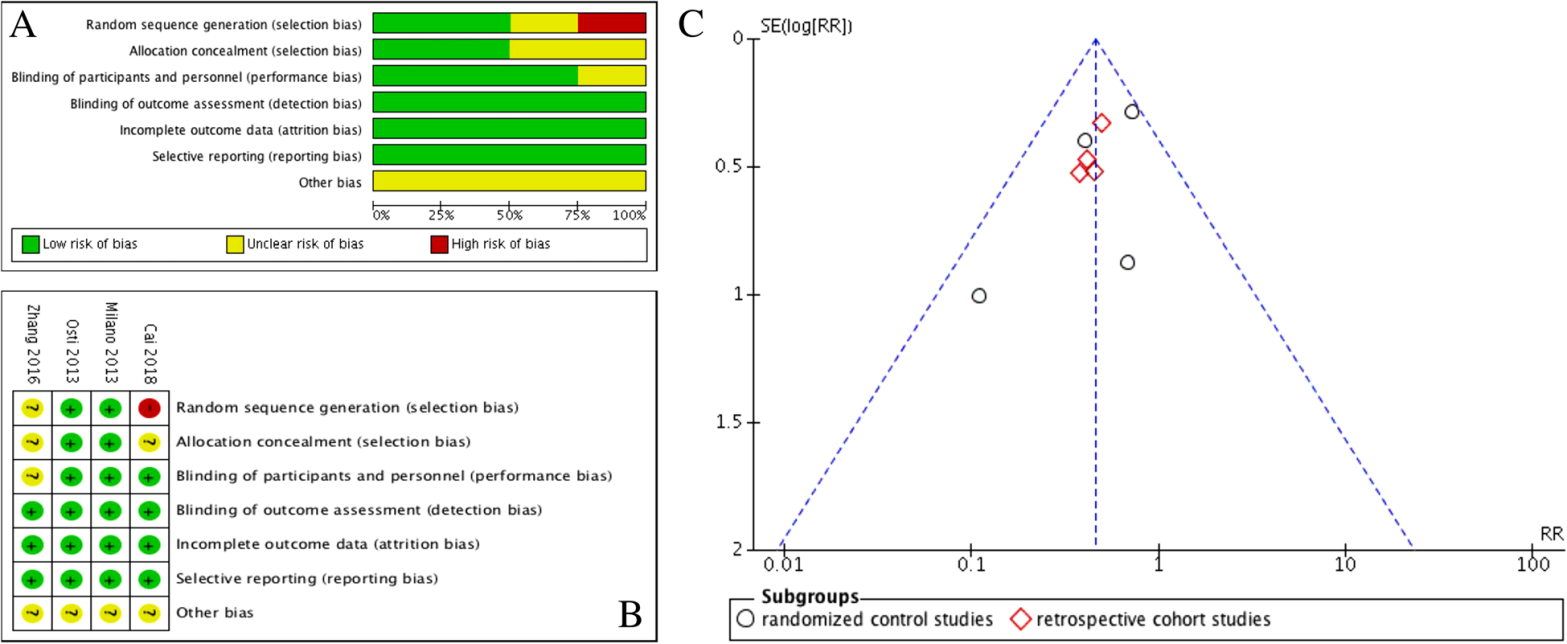
**a** Risk of bias graph exhibiting the review of the authors’ judgments about each risk of bias item presented as percentages across all included studies. **b** Risk of bias summary revealing the review of the authors’ judgments about each risk of bias item for included RCTs. Minus sign represents the risk of bias present, plus sign indicates the risk of bias absent, and question mark equals the risk of bias uncertain. **c** The funnel plots of the included studies. RR, relative risks; SE, standard error

The quality of the included studies was assessed according to the type of researches. As showed in Table 1, 4 retrospective cohort studies were evaluated by NOS with a total score ranged from 5 to 7. A bias assessment was applied to 4 RCTs (according to the Cochrane Handbook for Systematic Reviews of Interventions 5.0). The whole assessments were conducted by two reviewers separately, and any disagreement was resolved by the third reviewer. As showed in Figs. 2 and 2B, the quality of the included studies is high. Funnel plots demonstrated no visual evidence of publication bias (Fig. 2C).

### Meta-analysis results

#### Constant score and subgroup analysis

**Fig. 3:**
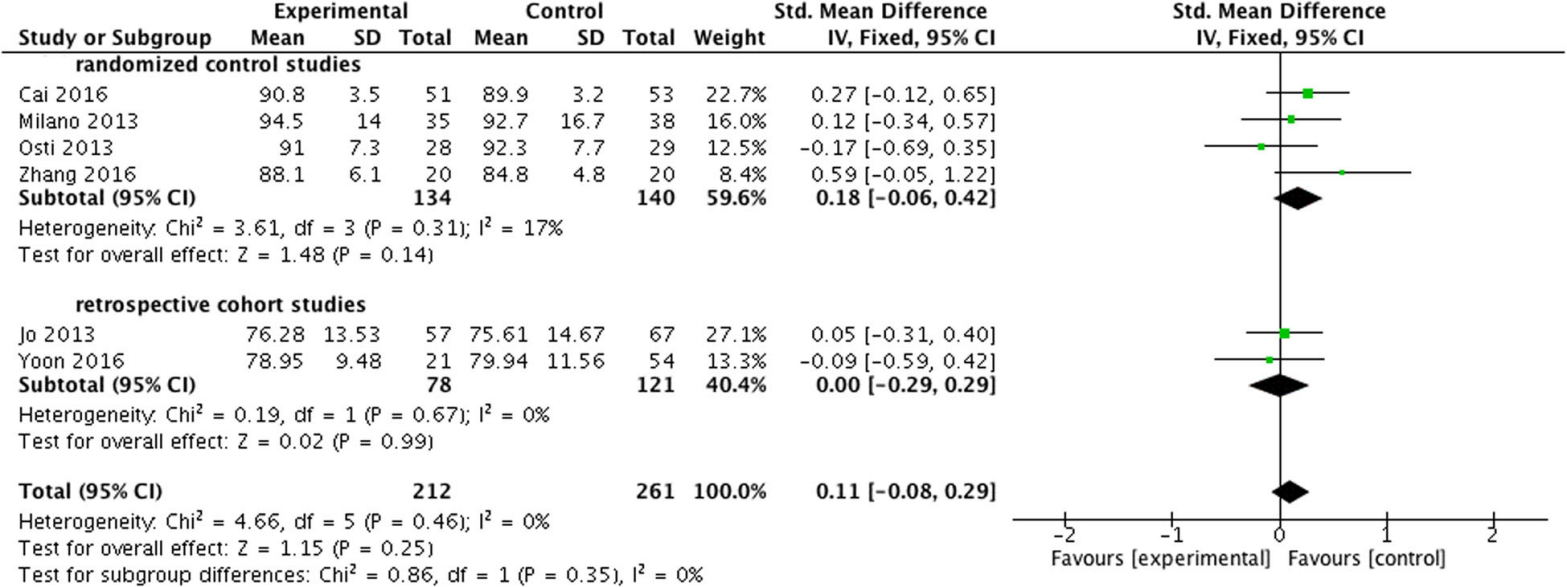
Difference in the Constant score and the subgroup analysis. CI, confidence interval; IV, inverse variance; SD, standard deviation. The solid squares indicate the mean difference and are proportional to the weights used in the meta-analysis. The solid vertical line indicates no effect. The horizontal lines represent the 95% CI. The diamond indicates the weighted mean difference, and the lateral tips of the diamond indicate the associated 95% CI

**Fig. 4:**
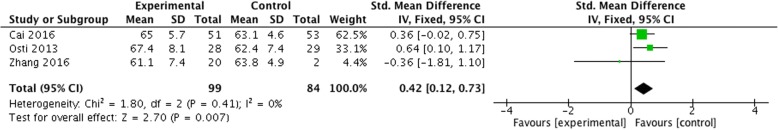
Difference in the Constant score at the third follow-up month. CI, confidence interval; IV, inverse variance; SD, standard deviation. The solid squares indicate the mean difference and are proportional to the weights used in the meta-analysis. The solid vertical line indicates no effect. The horizontal lines represent the 95% CI. The diamond indicates the weighted mean difference, and the lateral tips of the diamond indicate the associated 95% CI

The studies used different shoulder function scoring systems. Four RCTs [14, 15, 17, 18] and 2 retrospective cohort studies [16, 21] compared the postoperative Constant score between the two groups. Constant scores of the two groups were 75.61–92.7 and 76.28–94.5 respectively. There was no significant difference between the two groups (SMD = 0.11, 95% CI, − 0.08 to 0.29, *P* = 0.25, *I*^2^ = 0%) (Fig. 3). Among the above studies, 2 RCTs and 1 retrospective cohort study counted the Constant score after 3-month follow-up. The results showed that the Constant score of the BMS group was significantly higher than that of the control group at the third month after surgery (SMD = 0.42, 95% CI, 0.12 to 0.73, *P* = 0.007, *I*^2^ = 0%) (Fig. 4).

In addition, the subgroup analysis according to the type of the studies showed no significant difference in the Constant score between the two groups of both RCTs and retrospective cohort studies (SMD = 0.18, 95% CI, − 0.06 to 0.42, *P* = 0.14, *I*^2^ = 17%; SMD = 0.00, 95% CI, − 0.29 to 0.29, *P* = 0.99, *I*^2^ = 0%) (Fig. 3).

#### UCLA score and subgroup analysis

**Fig. 5:**
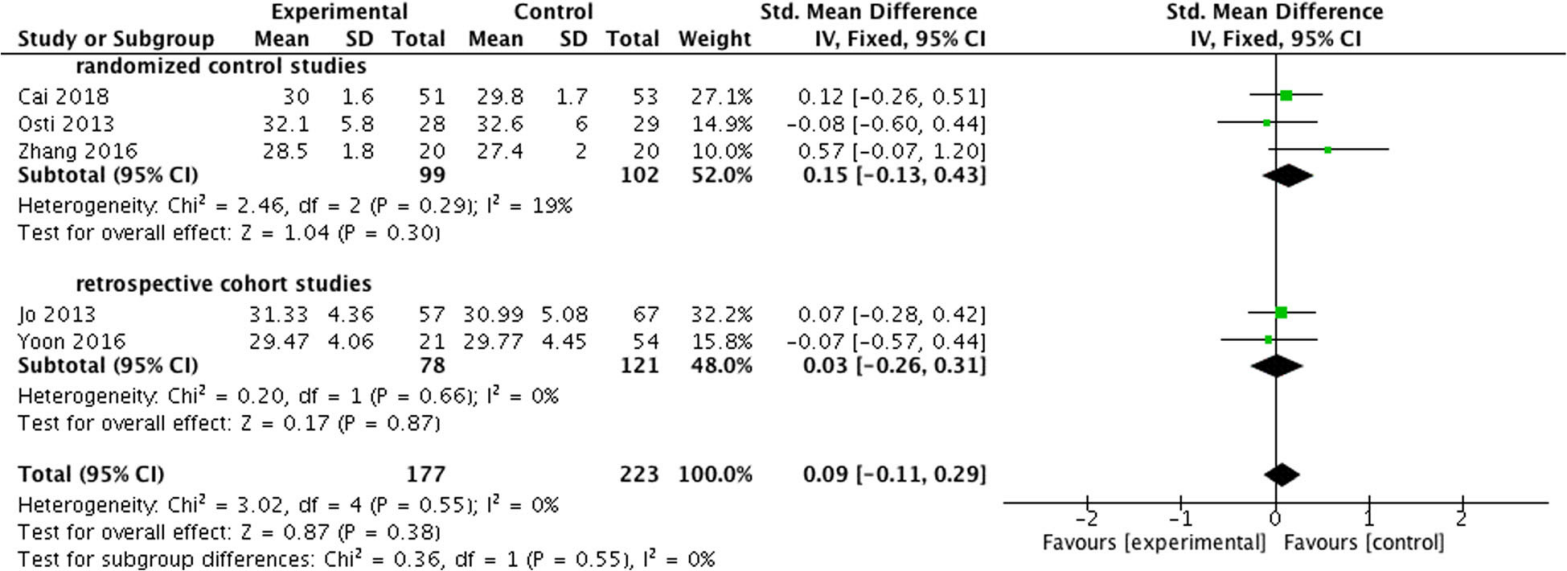
Difference in the UCLA score and the subgroup analysis. CI, confidence interval; IV, inverse variance; SD, standard deviation. The solid squares indicate the mean difference and are proportional to the weights used in the meta-analysis. The solid vertical line indicates no effect. The horizontal lines represent the 95% CI. The diamond indicates the weighted mean difference, and the lateral tips of the diamond indicate the associated 95% CI

Three RCTs [15, 17, 18] and 2 retrospective cohort studies [16, 21] compared the UCLA score after surgery between the two groups. UCLA score of the control group was 27.4–32.6, and that of the BMS group was 28.5–32.1. There was no significant difference in UCLA score between the two groups (SMD = 0.09, 95% CI, − 0.11 to 0.29, *P* = 0.55, *I*^2^ = 0%) (Fig. 5).

In addition, subgroup analysis according to the type of studies showed no significant difference in the UCLA score between the two groups of both RCTs and retrospective cohort studies (SMD = 0.15, 95% CI, − 0.13 to 0.43, *P* = 0.30, *I*^2^ = 19%; SMD = 0.03, 95% CI, − 0.26 to 0.31, *P* = 0.87, *I*^2^ = 0%) (Fig. 5).

#### Shoulder ROM

**Fig. 6:**
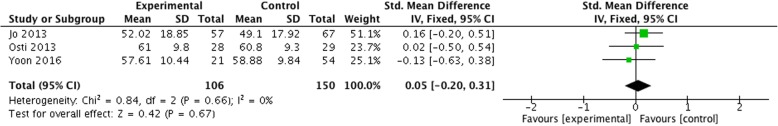
Difference in the external range of motion. CI, confidence interval; IV, inverse variance; SD, standard deviation. The solid squares indicate the mean difference and are proportional to the weights used in the meta-analysis. The solid vertical line indicates no effect. The horizontal lines represent the 95% CI. The diamond indicates the weighted mean difference, and the lateral tips of the diamond indicate the associated 95% CI

One RCT [15] and 2 retrospective cohort studies [16, 21] recorded and compared the shoulder ROM after surgery between the two groups. The motion was divided into two directions: external and forward ROM. External ROM of the control group was 49.1–60.8, while that of the experimental group was 52.02–61. There was no significant difference in the external ROM between the two groups (SMD = 0.05, 95% CI, − 0.20 to 0.31, *P* = 0.67, *I*^2^ = 0%) (Fig. 6).

**Fig. 7:**
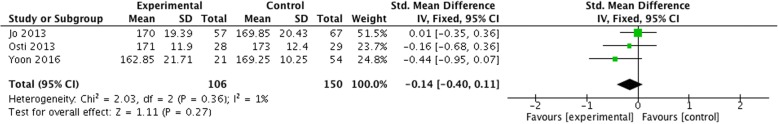
Difference in the forward range of motion. CI, confidence interval; IV, inverse variance; SD, standard deviation. The solid squares indicate the mean difference and are proportional to the weights used in the meta-analysis. The solid vertical line indicates no effect. The horizontal lines represent the 95% CI. The diamond indicates the weighted mean difference, and the lateral tips of the diamond indicate the associated 95% CI

The forward ROM was 169.25–173 in the control group and 162.85–171 in the experimental group. There was also no significant difference in the forward ROM between the two groups (SMD = 0.00, 95% CI, − 1.20 to 1.20, *P* = 1.00, *I*^2^ = 0%) (Fig. 7).

#### VAS, SST, and ASES scores

**Fig. 8:**
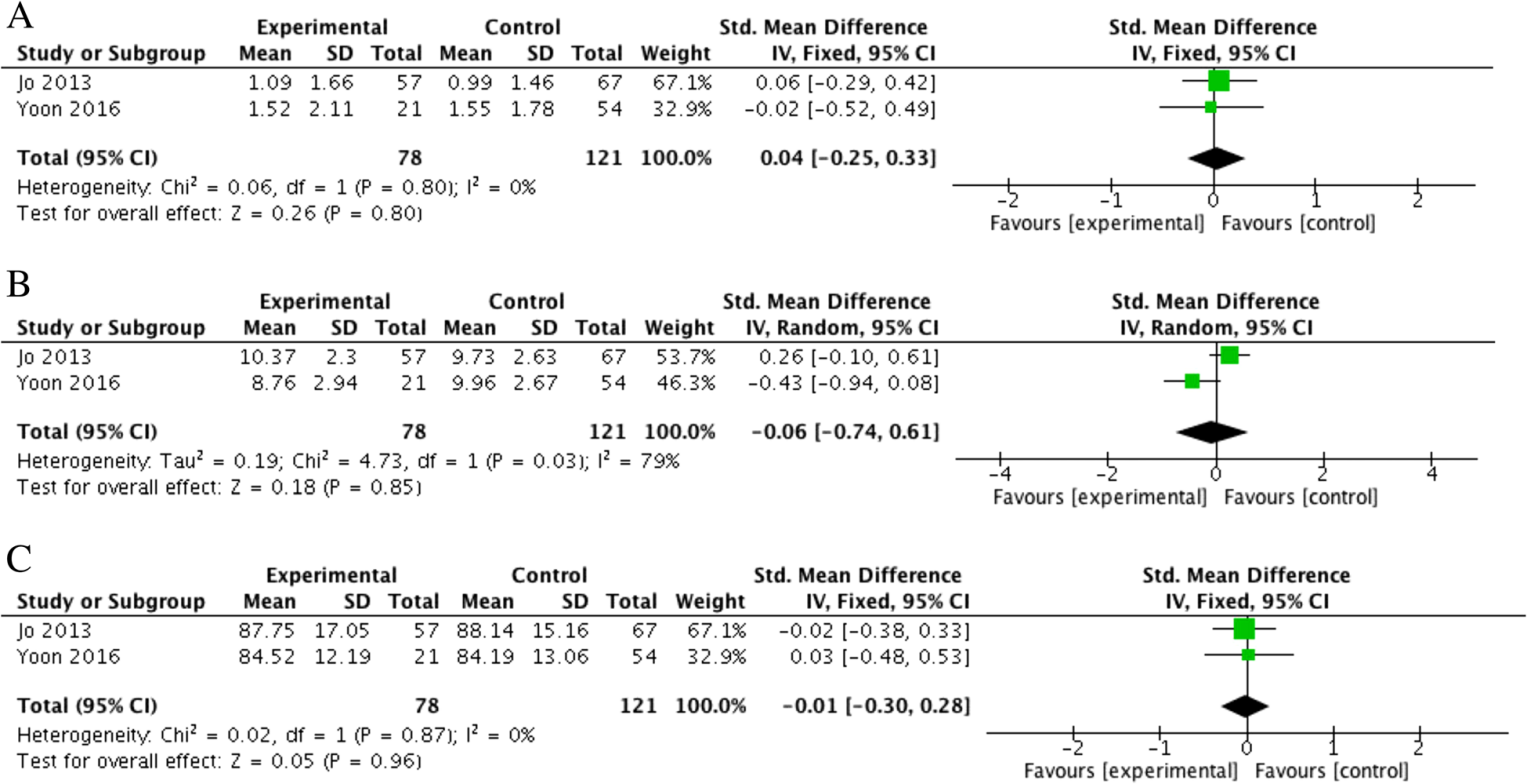
**a** Difference in the VAS. **b** Difference in the SST score. **c** Difference in the ASES score. CI, confidence interval; IV, inverse variance; SD, standard deviation. The solid squares indicate the mean difference and are proportional to the weights used in the meta-analysis. The solid vertical line indicates no effect. The horizontal lines represent the 95% CI. The diamond indicates the weighted mean difference, and the lateral tips of the diamond indicate the associated 95% CI

Two retrospective cohort studies [16, 21] recorded and compared the VAS, SST, and ASES scores after surgery in both groups. The scores of the control group were 0.99–1.55, 9.73–9.96, and 84.19–88.14, while those of the experimental group were 1.09–1.52, 8.73–10.37, and 84.52–87.75, respectively. There was no significant difference in VAS, SST, or ASES scores between the two groups (SMD = 0.04, 95% CI, − 0.25 to 0.33, *P* = 0.80, *I*^2^ = 0%; SMD = 0.06, 95% CI, − 0.74 to 0.61, *P* = 0.85, *I*^2^ = 79%; SMD = 0.01, 95% CI, − 0.30 to 0.28, *P* = 0.96, *I*^2^ = 0%) (Figs. 8A, B, and C).

#### The re-tear rate and subgroup analysis

**Fig. 9:**
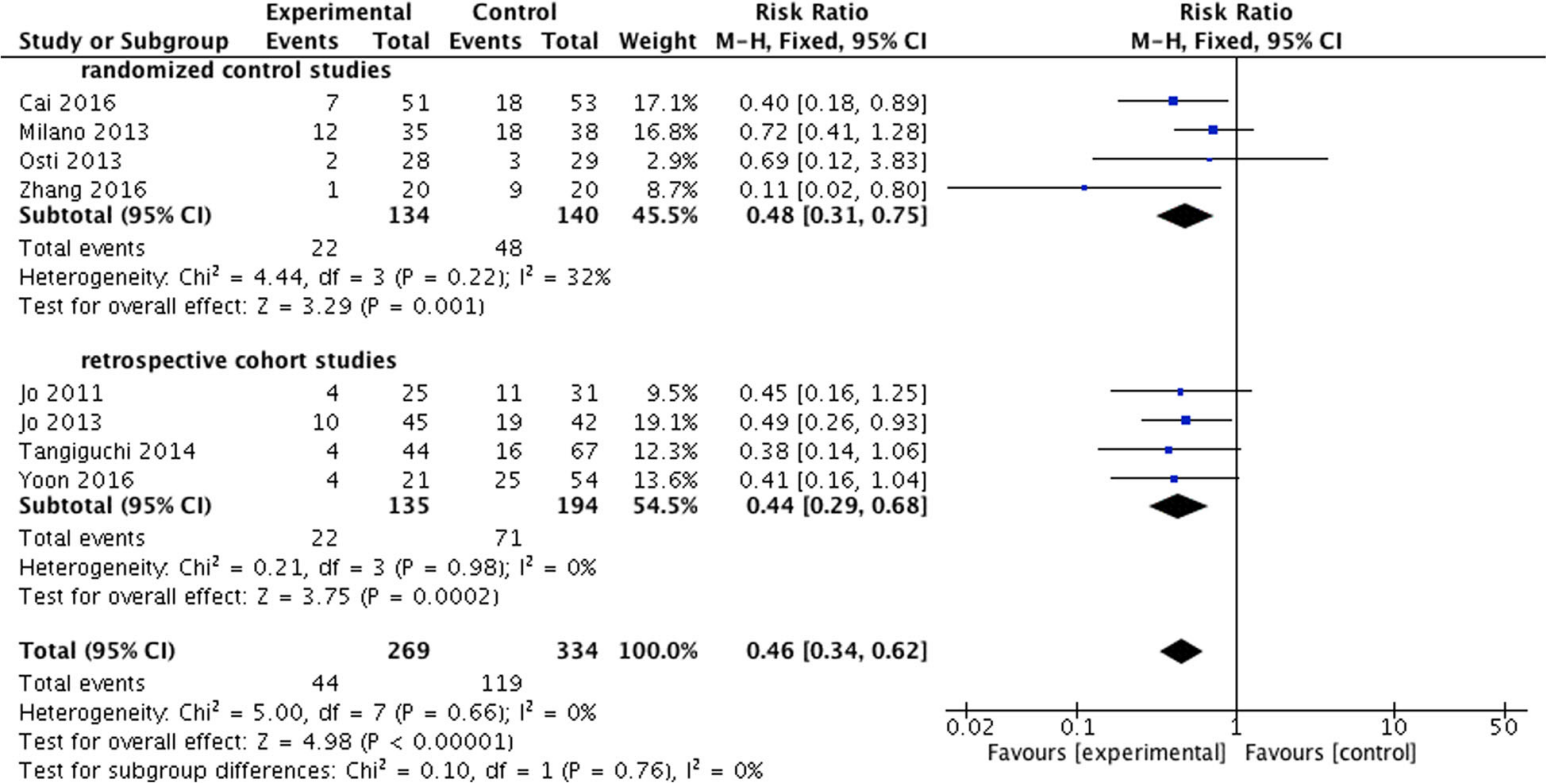
Difference in the incidence of re-tear and the subgroup analysis. CI, confidence interval; M-H, Mantel-Haenszel. The solid squares indicate the mean difference and are proportional to the weights used in the meta-analysis. The solid vertical line indicates no effect. The horizontal lines represent the 95% CI. The diamond indicates the weighted mean difference, and the lateral tips of the diamond indicate the associated 95% CI

The rates of rotator cuff re-tear in both groups were reported in all 8 articles, including 4 RCTs [14, 15, 17, 18] and 4 retrospective cohort studies [16, 19–21] (*n* = 640). One hundred nineteen of 334 patients were re-tear in the control group, accounting for 35.6% of the total. Forty-four of 269 patients were re-tear in the experimental group, accounting for 16.4% of the total. There was a significant difference between the two groups. The rate of the experimental group was significantly lower than that of the control group (RR = 0.46, 95% CI, 0.34 to 0.62, *P* < 0.00001, *I*^2^ = 0%) (Fig. 9).

Besides, the subgroup analysis showed that of the RCTs (*n* = 274), the re-tear rate was 34.3% (48/140) in the control group and 16.4% (22/134) in the experimental group. There was a significant difference between the two groups (RR = 0.48, 95% CI, 0.31 to 0.75, *P* = 0.001, *I*^2^ = 32%). Of retrospective cohort studies (*n* = 329), the rate was 36.6% (71/194) in the control group and 16.3% (22/135) in the experimental group. There was a significant difference between the two groups (RR = 0.44, 95% CI, 0.29 to 0.68, *P* = 0.0002, *I*^2^ = 0%) (Fig. 9).

## Discussion

This meta-analysis evaluated the efficacy of applying the BMS to arthroscopic rotator cuff neoplasty. The result showed that the re-tear rate of the experimental group was lower than that of the control group after at least 1 year’s follow-up. However, the result of clinical function had no significant differences, except that the researches by Osti et al. [15, 17, 18] confirmed that the Constant score, the UCLA score, and the ROM of the patients with BMS were all significantly improved and pain declined within 3 months after the surgery. However, the above scores of the two groups became no difference after 1 to 2 years’ follow-up.

With the development of rotator cuff pathology, researchers gradually gain a better understanding of the complicated healing process of the tendon-to-bone interface. The position for the tendon to insert into the bone is comprised of highly specialized tissue, which consists of four layers: tendon, fibrocartilage, calcified cartilage, and bone. The cartilage plays the role of buffering concussion and reducing the stresses so as to transfer the muscular stretch from the tendon to the bone. However, the fibrocartilage layer of normal tendon-to-bone structure always is often unable to regenerate after the rotator cuff neoplasty, which is only filled up with the scar tissue formed by the collagen fiber. Therefore, the tensile strength is largely reduced, and re-tear occurs more easily under stress with normal intensity [23].

The stem cells have huge potential in repairing the rotator cuff injury, which can differentiate towards the tendon tissue to achieve the purpose of repairing when stimulated by the endogenous or exogenous stress. The different-source-derived stem cells have increasingly played an important role in the healing of tendon-to-bone interface, including the bone marrow mesenchymal stem cells (BMSCs), and adipose-derived stem cell (ASC). Ouyang showed that the allogeneic BMSCs transplanted to the patellar tendon of the rabbit were differentiated into tendon-like cells 5 weeks later [24]. Chong found that injecting the BMSCs in the early period of tendon injury could effectively promote the healing and improve its biological mechanical property [25]. Beitzel made a retrospective analysis on applying the BMSCs to repair the rotator cuff injury. Seven articles demonstrated it could promote the healing and 1 showed no significant efficacy [26].

With the development of arthroscopic technology, some scholars have studied to apply the BMSCs during the arthroscopic repairing of rotator cuff injury. Hernigou injected the concentrated BMSCs into the tendon-to-bone interface, and after a 24-month follow-up, they found that the healing rate of the experimental group was significantly higher compared with the control group (100% vs 67%) and the re-tear rate was significantly lower (13% vs 56%) [27]. Yokoya applied the BMSCs combined with biological absorbable materials and demonstrated that BMSCs can increase the secretion of collagen I and enhance the mechanical strength of the regenerated rotator cuff [28].

The clinical application of the BMSCs has been limited by such factors as the following: (1) large cost and time consumption for BMSCs extraction, culture, and differentiation; (2) possible complications of severe pain and infection caused by bone marrow aspiration before the surgery; and (3) gradual decrease of BMSC quantity with aging and so on [29]. Thus, the “crimson duvet,” one of BMS technology, was proposed by Snyder in 2009. He suggested drilling at the trochiter under arthroscopy to overflow the BMSCs and growth factors from the marrow cavity and repairing the rotator cuff routinely at the same time, which could improve the biological repair of the tendon-to-bone interface. This is a simple technology without the need for any special instrument, an increase of any additional injury or complication, showing its obvious advantages. Although there is little evidence for the efficacy of this technology. The latest related researches on rotator cuff injury model indeed have shown that BMSCs can effectively promote the tendon-to-bone healing. Mazzocca obtained the bone marrow from the anchor and separated it to confirm the existence of BMSCs, which have the osteogenic potential and can promote the healing of rotator cuff injury [30]. Kida drilled at the trochiter and observed that BMSCs could adhere to the tendon-to-bone interface via the holes of footprint and better promote the healing of the rotator cuff so as to improve its fixation strength [12].

However, this study showed that the application of BMS has no advantage over the treatment without BMS in the aspect of clinical outcomes. On the other hand, some patients with re-tear after healing might not show any clinical manifestation, such as severe pain or obvious dysfunction, or were just accompanied with weakened strength of the shoulder. They reported that 51% symptomless patients with rotator cuff re-tear would gradually show clinical symptoms 2.8 years later [31]. While only the researches by Osti and Jo [15, 16] met the follow-up time in this study. In the meanwhile, our result displayed that the BMS delivered a satisfactory short-term efficacy within 3 months after the surgery. Thus, we cannot hastily conclude that BMS cannot improve the clinical outcome of patients. The score of clinical outcome cannot entirely substitute the efficacy of BMS, while the regular and long-term follow-up is also very important.

Some clinical factors will impact the healing of the rotator cuff. One of the problems in BMS is the absence of a standard process. Different studies adopted different strategies. This meta-analysis reported the instruments, process, and the corresponding location of BMS in these studies (Table 2). The subgroup analysis showed that BMS groups in four retrospective cohort studies had a significant decline in postoperative re-tear rate compared with the control. BMS technologies they used were similar: using osteotome with 2.1 mm diameters to drill on the region from the edge of the cartilage defect site to the lateral border of trochiter at an interval of 4–5 mm and depth of 10 mm. But this might be not the best plan. Some defects exist when BMS treatment is conducted in footprint, such as uneven distribution of bone marrow. The treatment will be a failure if the drilling density in the unit area is too small, while too large density will significantly damage the cancellous bone of trochiter, which is not favorable for bone remodeling. In addition, the drilling being too near the anchor area is prone to reduce the fixation intensity of the anchor. Thus, Cai and Zhang improved the BMS technology by acquiring high drilling density using the diameter of 0.5 mm [17, 18]. This method has an advantage of drilling at multiple points to widen and homogenize the distribution of MSCs in footprint and minimize the damage to the cancellous bone. Yet this makes MSCs to be drained from the footprint more easily.

This study has some limitations: Firstly, 4 of 8 articles selected in our meta-analysis are RCT research, while the rest are retrospective cohort research. Although it has been found via the subgroup analysis that the final results of different types of research are almost the same, yet this will undoubtedly increase the selection bias of this study. Secondly, the included clinical researches have certain heterogeneity: single-row or double-row repair, different sizes of tear, and different schemes adopted for BMS. Thirdly, lack of literature has hindered us in sufficient subgroup analysis, including the position, size, time, fatty infiltration of the tear.

## Conclusions

BMS therapy in full thickness rotator cuff repair showed no statistically significant difference compared with conventional therapy in clinical outcomes; while tendon-to-bone healing was better in patients with BMS. Further, more randomized controlled studies with BMS, with longer follow-up time, may eventually show enhanced clinical outcomes based on better healing rates.

## Abbreviations

ASCs: Adipose-derived stem cell
ASES: American Shoulder and Elbow Surgeons
BMS: Bone marrow stimulation
BMSCs: Bone marrow mesenchymal stem cells
MSCs: Mesenchymal stem cells
NOS: Newcastle-Ottawa Scale
PRP: platelet-rich plasma
RCT: Randomized controlled trial
ROM: Range of motion
SST: Simple Shoulder Test
UCLA: University of California at Los Angeles
VAS: Visual analogue scale
